# An Adaptive Multi-Population Approach for Sphericity Error Evaluation in the Manufacture of Hemispherical Shell Resonators

**DOI:** 10.3390/s24051545

**Published:** 2024-02-28

**Authors:** Dongfang Zhao, Junning Cui, Xingyuan Bian, Zhenghao Li, Yanxu Sun

**Affiliations:** 1 Center of Ultra-Precision Optoelectronic Instrument Engineering, Harbin Institute of Technology, Harbin 150080, China; 20b901006@stu.hit.edu.cn (D.Z.); bianxingyuan@hit.edu.cn (X.B.); 23s001024@stu.hit.edu.cn (Z.L.); 22b901030@stu.hit.edu.cn (Y.S.); 2 Key Lab of Ultra-Precision Intelligent Instrumentation (Harbin Institute of Technology), Ministry of Industry and Information Technology, Harbin 150080, China

**Keywords:** hemispherical shell resonator, spherical error, multi-population, high-precision evaluation

## Abstract

The performance of a hemispherical resonant gyroscope (HRG) is directly affected by the sphericity error of the thin-walled spherical shell of the hemispherical shell resonator (HSR). In the production process of the HSRs, high-speed, high-accuracy, and high-robustness requirements are necessary for evaluating sphericity errors. We designed a sphericity error evaluation method based on the minimum zone criterion with an adaptive number of subpopulations. The method utilizes the global optimal solution and the subpopulations’ optimal solution to guide the search, initializes the subpopulations through clustering, and dynamically eliminates inferior subpopulations. Simulation experiments demonstrate that the algorithm exhibits excellent evaluation accuracy when processing simulation datasets with different sphericity errors, radii, and numbers of sampling points. The uncertainty of the results reached the order of 10^−9^ mm. When processing up to 6000 simulation datasets, the algorithm’s solution deviation from the ideal sphericity error remained around −3 × 10^−9^ mm. And the sphericity error evaluation was completed within 1 s on average. Additionally, comparison experiments further confirmed the evaluation accuracy of the algorithm. In the HSR sample measurement experiments, our algorithm improved the sphericity error assessment accuracy of the HSR’s inner and outer contour sampling datasets by 17% and 4%, compared with the results given by the coordinate measuring machine. The experiment results demonstrated that the algorithm meets the requirements of sphericity error assessment in the manufacturing process of the HSRs and has the potential to be widely used in the future.

## 1. Introduction

The hemispherical resonator gyroscope (HRG) is the most accurate inertial sensor in the world [1,2,3,4,5]. In recent years, the demand for HRGs has significantly increased in aerospace and deep space exploration [6,7,8,9]. The hemispherical shell resonator (HSR) is the core component of the HRG and its main working area is the hemispherical thin-walled spherical shell structure. During the manufacturing process, the inevitable sphericity error can cause uneven mass distribution and elastic modulus in thin-walled spherical shells, which can affect the accuracy of HRGs. In addition, shaping and manufacturing the HSR requires multiple processes, such as rough machining, precision grinding, and precision polishing [10,11]. Sphericity error is a critical technical indicator used to assess the quality of thin-walled spherical shells during each process. Different processes have varying requirements for sphericity error. Accurately evaluating the sphericity error in thin-walled spherical shells is crucial for achieving the high-precision manufacturing of HRGs [12].

To strictly control the sphericity error in thin-walled spherical shells, it is necessary to use form error measurement equipment to measure and evaluate sphericity error [13,14,15,16,17,18,19]. Form error measurement equipment, such as a coordinate measuring machine (CMM), typically saves the sampled shell contour data as a large dataset in XYZ coordinate form [20]. The International Organization for Standardization (ISO) and the American Society of Mechanical Engineers (ASME) standards encompass four criteria for assessing sphericity error: the least squares criterion, maximum inscribed criterion, minimum circumscribed criterion, and minimum zone criterion [21,22]. In order to control the mass distribution of the thin-walled spherical shells, the minimum zone criterion is the most suitable method for evaluating the sphericity error of the HSRs. However, the ISO and ASME standards lack explicit methodologies for implementing the minimum zone criterion [23,24,25]. Furthermore, evaluating this dataset based on the minimum zone criterion involves non-differentiable and unconstrained problems [26,27,28]. Meeting the requirements for speed, accuracy, and robustness in the HSR manufacturing process presents a significant challenge for algorithms that evaluate sphericity error based on the minimum zone criterion.

In order to address the challenge of sphericity error evaluation based on the minimum zone criterion, Chen et al. [29] established four concentric sphere models by solving systems of linear algebraic equations and selected the one with the minimum sphericity error as the final evaluation result. This method laid the foundation for approaches based on selecting sample points [12]. Fan et al. [30] proposed a minimum potential energy method inspired by this method. At the same time, Fei Liu et al. [31] designed a method for evaluating sphericity error using chord relationships among sample points. As this type of method requires searching and traversing all sampling points to select combinations that meet the minimum zone criterion, when the volume of data is relatively small, the method of selecting sampling points exhibits high accuracy and robustness. However, when measuring and evaluating the HSRs, the required amount of data increases dramatically, leading to a pronounced surge in the computational cost and decreasing robustness for the algorithms based on selecting sampling points. Therefore, these methods are unsuitable for evaluating sphericity errors in the manufacturing process of the HSRs.

With the development of heuristic algorithms [32,33,34,35], they are increasingly being used for sphericity error evaluation. Jiang et al. [36] proposed using a cuckoo search algorithm for evaluating sphericity error, while Lei et al. [37] introduced a geometric optimization search-based sphericity error evaluation algorithm. Compared with the method of selecting sample points, the solution accuracy and robustness of the heuristic algorithm are less dependent on the amount of sampled data, and the solution results depend more on the design of the algorithm itself. Moreover, heuristic algorithms are directly applied to the assessment of the HSR’s sphericity errors without the integration of specialized search strategies informed by domain knowledge. In that case, there is a risk of compromising their solution’s accuracy and robustness. After incorporating domain-specific search strategies, these techniques can potentially be used to assess sphericity errors in the manufacturing process of the HSRs. Furthermore, heuristic algorithms based on multi-populations have recently experienced notable advancements. The collaboration of diverse subpopulations enhances heuristic algorithms’ search capabilities [38,39,40]. However, no researcher has yet applied this method to the field of sphericity error assessment.

The manufacturing process of the HSRs involves multiple processes, and the field of inertial navigation has an immense demand for the most accurate inertial sensor. Therefore, in the HSRs’ manufacturing process, the evaluation of their sphericity error exhibits characteristics of large data volume, high speed, high precision, and robustness. Current methods for assessing sphericity error do not meet the demands of this scenario. After analyzing the distribution characteristics of sphericity error, we propose a novel adaptive multi-population method to effectively assess high-precision sphericity error in the manufacturing process of the HSRs. The main contributions of this study can be summarized as follows:

1. For the specific needs of the HSR production, this study explores the spatial distribution characteristics of the sphericity error. The sphericity error gradient has been observed to be larger, and the characteristics are more concise at locations farther from the ideal sphere center. In contrast, the sphericity error changes are complicated in the region close to the ideal sphere center.
2. Based on the in-depth analysis of the sphericity error distribution characteristics, we design an adaptive multi-population cooperative search algorithm. The algorithm guides the subpopulation individuals to search through the global optimal solution and subpopulation optimal solution, and periodically reorganizes the subpopulations and eliminates the inferior subpopulations. This not only achieves fast convergence at the beginning of the search but also enables a detailed search later when the region near the ideal sphere center is approached.
3. The proposed algorithm’s accuracy and robustness are verified through numerous experiments, proving that it can effectively meet the needs of HSR production. This algorithm can significantly improve the accuracy of the existing form error measurement equipment in data processing.

## 2. Methods

### 2.1. Sphericity Error Evaluation Model and Distribution Characteristics

#### 2.1.1. Mathematical Model of Minimum Zone Criteria

**Figure 1:**
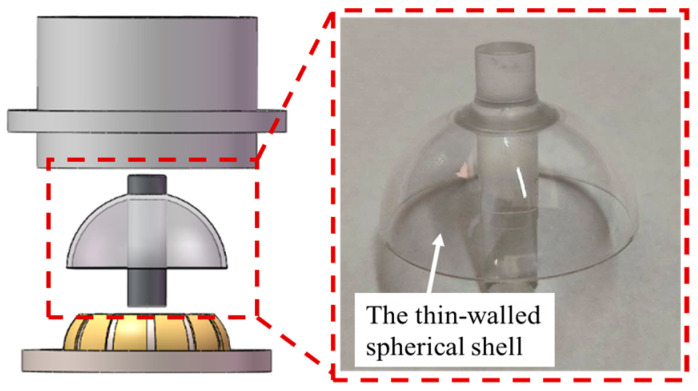
Schematic diagram of the hemispherical resonator gyroscope.

The non-uniform mass distribution of a thin-walled spherical shell significantly impacts the HSR’s Q factor and vibration characteristics. Compared to other criteria given by ASME and ISO standards [21,22], such as the least squares criterion, maximum inscribed criterion, and minimum circumscribed criterion, evaluating the sphericity error based on the minimum zone criteria is more suitable for ensuring the quality control of the HSRs [11,41]. Figure 1 shows the schematic diagram of the hemispherical resonator gyroscope and provides a real picture of the hemispherical shell resonator.

For the thin-walled spherical shell profile dataset collected by the form error measurement equipment, the method of assessing the sphericity error based on the minimum zone criteria is to find two concentric spheres encompassing the entire measured profile and to minimize the difference in radius between the two concentric spheres. The sphericity error objective function based on the minimum zone criterion can be expressed as Equation (1). In this process, the central task is to find an ideal spherical center $p_{0}^{*}$ that minimizes the sphericity error *S* of the objective function.

(1) $$S=\min\left\{max{\left\|p_{i}-p_{0}\right\|}_{2}-min{\left\|p_{i}-p_{0}\right\|}_{2}\right\}$$

where $p_{0}$ represents the center coordinate of the sphere, and $p_{i}$ represents the set of sampled point coordinates, where *i* is the number of sampled points.

#### 2.1.2. Sphericity Error Spatial Distribution Characteristics

**Figure 2:**
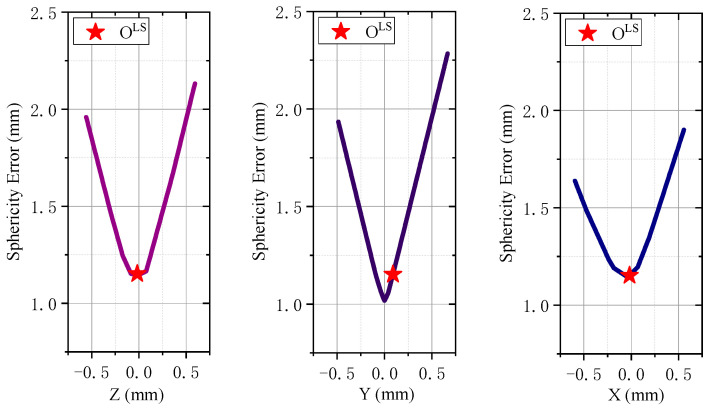
The sphericity error variation curves along the X, Y, and Z directions are presented, with the origin located at the least-squares spherical center. The original data used for drawing were obtained from reference [29].

It is widely acknowledged that the sphericity error varies at different rates depending on the position [42,43]. To demonstrate this, we use the spherical contour sampling dataset from reference [29] and apply the least squares method to obtain the initial spherical center. And the optimal solution is located close to the least squares spherical center. Therefore, we plot the variations of sphericity error along the X, Y, and Z directions of the least squares spherical center, as shown in Figure 2. The graph reveals that the decrease in sphericity error rate is steeper farther away from the least squares solution, and there are potential locally optimal solutions around the ideal solution. The potential locally optimal solutions exist because the sphericity error depends on the sampling points, and abrupt changes in these points can lead to sudden shifts in the sphericity error.

From Figure 2, it is evident that in the vicinity of the least squares spherical center, the variation in sphericity error is complex. Conversely, when moving away from the least squares spherical center, the gradient of sphericity error variation is substantial, indicating a pronounced downward trend. 

Capitalizing on this distinctive characteristic, we formulated a multi-population search algorithm and employed clustering methods to initiate subpopulations, periodically recombining the subpopulations. Guided by both the global optimal solution and the optimal solution within each subpopulation, most subpopulations swiftly converged to the vicinity of the ideal spherical center in the early stages of the search. During the search process, we designed an elimination mechanism, where subpopulations with inadequate individuals that cannot be clustered are eliminated, and population individuals converge to the dominant subpopulations. In the later stages of the search, while subpopulations converge to the ideal spherical center vicinity, the number of subpopulations gradually decreases while the number of individuals in the remaining subpopulations increases, thus achieving a detailed search near the ideal spherical center. In the manufacturing process of the HSRs, we can use the above mechanism to achieve a sphericity error assessment that meets the requirements of speed, precision, and robustness.

### 2.2. Proposed Method

**Figure 3:**
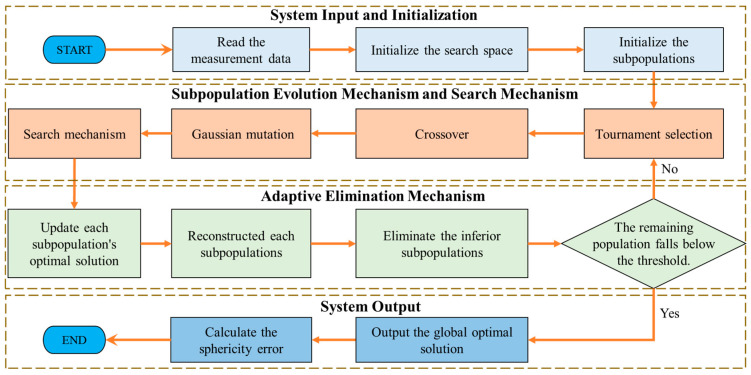
Schematic diagram of the algorithm flow.

The algorithmic framework designed for this study is illustrated in Figure 3. Initially, a large population is randomly split into various subpopulations for the purpose of initialization. Subsequently, each subpopulation conducts evolution and search processes, with periodic updates made to both the global optimal solution and the optimal solution specific to the subpopulation. Following this, the subpopulations are recombined based on clustering methods, utilizing the optimal solution of each subpopulation as the center. Meanwhile, inferior subpopulations are adaptively eliminated. The search stops when the remaining subpopulation falls below a specified threshold, and the global optimal solution is the output of the center of the concentric spheres.

#### 2.2.1. Initialization Process

(1) Initializing the Search Space

Establishing an appropriate search space ***R*** is crucial for enhancing search efficiency. The minimum zone solution is commonly considered to be contained within a sphere. The center of the sphere corresponds to the least squares solution, and the radius is determined by the least squares sphericity error [44]. Therefore, the search space ***R***, initial spherical center $\left(x_{0},y_{0},z_{0}\right)$, and initial sphericity error $S_{0}$ are initialized based on the least squares method.

A linear system of equations can be constructed from a set of sampled point coordinates $p_{i}={\left[x_{i},y_{i},z_{i\;}\right]}^{T}\in S$, where $i=\left(1,2,\ldots ,n\right)$, as shown in Equation (2),

(2) Ax=b

where $A={\left[p_{1},p_{2},\ldots {,p}_{n},I\right]}^{T}$, $b={\left[{\left\|p_{1}\right\|}_{2}^{2},{\left\|p_{2}\right\|}_{2}^{2},\ldots ,{\left\|p_{n}\right\|}_{2}^{2}\right]}^{T}$, $I={\left[1,1,\ldots ,1\right]}^{T}$. Equation (3) can be obtained by transforming the system of equations into regular equations.

(3) $$A^{T}Ax=A^{T}b$$

By solving Equation (3), the least squares spherical center coordinates $O_{LS}\left(x_{LS},y_{LS},z_{LS}\right)$ can be obtained. Then, the initial sphericity error $S_{0}$ can be defined based on the minimum zone criterion.

After obtaining the initial spherical center and the initial sphericity error $O_{LS}\left(x_{LS},y_{LS},z_{LS}\right)$, the spatial position of the minimum zone spherical center $O_{MZ}\left(x_{MZ},y_{MZ},z_{MZ}\right)$ can be determined by the formulation presented in Equation (4). Equation (4) serves as a representation of the search space ***R*** and defines the boundary conditions.

(4) $$R:\left\{\begin{array}{c} x_{LS}-S_{0}\;\leq \;x_{MZ}\;\leq \;x_{LS}+S_{0}\;\\ y_{LS}-S_{0}\leq \;y_{MZ}\;\leq \;y_{LS}+S_{0}\\ z_{LS}-S_{0}\leq \;z_{MZ}\;\leq \;z_{LS}+S_{0} \end{array}\right.$$

(2) Initializing the Subpopulations

Within this search space ***R***, an initial population ${Pop}_{i}={\left[x_{i},y_{i},z_{i\;}\right]}^{T}\in R$,$\;i=\left(1,2,\ldots ,n\right)$ can be generated. After obtaining the initial population Pop, *K* individuals $P_{ai}=P_{a1},P_{a2},..,P_{aK}$ are randomly selected as cluster centers. The Euclidean distance ${\left\|{Pop}_{i}-P_{ai}\right\|}_{2}$ between each individual ${Pop}_{i}$ in the initial population Pop and the cluster center $P_{ai}$ is calculated. Then, each individual ${Pop}_{i}$ in the population is assigned to the subpopulation *subPop*1, *subPop*2, …, *subPopK*. corresponding to the nearest cluster center $P_{ai}$. By using clustering methods for subpopulation initialization, the initialized subpopulations are distributed in non-overlapping local regions of the search space ***R***. This step completes the initialization of the algorithm. And the pseudocode describing the subpopulation initialization based on clustering methods is as Algorithm 1 follows.
**Algorithm 1: Initialize the Subpopulations Based on Clustering****Input:**-Search space ***R***-The number of subpopulations *K*-Initial population **Pop**-$\mathrm{Initial}\;\mathrm{population}\;\mathrm{individual}\;{Pop}_{i}$-$\mathrm{Cluster}\;\mathrm{centers}\;P_{a1},P_{a2},..,P_{aK}$**Output:**-Subpopulations *subPop1*, *subPop2*, *…*, *subPopK*
1: Generate the initial population **Pop** within the search space ***R***; 2: Randomly select *K* individuals $P_{a1},P_{a2},..,P_{aK}$ as cluster centers; 3: **for** each ${Pop}_{i}$ in **Pop**: 4:  Calculate the Euclidean distance ${\left\|{Pop}_{i}-P_{ai}\right\|}_{2}$ between ${Pop}_{i}$ and each cluster center $P_{ai}$; 5:  Assign ${Pop}_{i}$ to the subpopulation subPopi corresponding to the nearest cluster center $P_{ai}$; 6: **end for** 7: **Return** *subPop1*, *subPop2*, *…*, *subPopK*

#### 2.2.2. Search Mechanism

(1) Subpopulation Evolution Mechanism

Before each subpopulation starts searching, it undergoes a preliminary independent evolution to ensure the diversity of the subpopulation. The evolutionary search process is as follows.

Firstly, a tournament selection method is used to select individuals. Then, two individuals are randomly selected with equal probability and compared based on their sphericity error. The individual with the smaller sphericity error is selected to enter the next generation subpopulation, until the new subpopulation size reaches the original size. The optimal solution of the subpopulation is directly retained for the next generation.

Secondly, two parents are selected with equal probability for uniform crossover operation, generating two new offspring. The offspring with the smaller sphericity error is selected to be retained in the next generation subpopulation.

Finally, we can use Gaussian mutation as a means of mutating individuals in the population. We select individuals from outside the optimal solution of the subpopulation with a given probability and apply perturbations based on Gaussian mutation. The individuals of the subpopulation are perturbed based on the Gaussian mutation probability shown in Equation (5). Before perturbation, the *d*-dimensional component size of the subpopulation *j* is ${subPopK}_{jd}^{i}$, which after perturbation becomes ${subPopK}_{jd}^{i+1}$. In Equation (5), *σ* represents the standard deviation, *i* represents the generation number, and *j* represents the individual number within the subpopulation.

(5) $$f\left({subPopK}_{jd}^{i+1}\right)=\frac{1}{\sqrt{2\pi }\sigma }\exp\left(\frac{-{\left(subPopK_{jd}^{i+1}-subPopK_{jd}^{i}\right)}^{2}}{2\sigma^{2}}\right)$$

(2) Subpopulation Search Mechanism

We introduce the concepts of the subpopulation optimal solution (pbest) and the global optimal solution (gbest) in the traditional particle swarm algorithm. The pbest refers to the optimal solution within a specific subpopulation, while the pbest is the optimal solution among all subpopulations. During the search process, we update the global optimal solution (gbest) and the subpopulation optimal solution (pbest) in real time. The detailed search strategy includes position and velocity update formulas; the position update formula is shown in Equation (6), and the velocity update formulas are shown in Equations (6) and (7). In the search process, we will execute this part of the content several times until it reaches the predetermined number of times ***N_iter_***.

(6) $$subPopK_{j}^{i+1}=subPopK_{j}^{i}+V_{j}^{i+1}$$

(7) $$\begin{array}{c} V_{j}^{i+1}=c_{1}\cdot V_{j}^{i}+c_{2}\cdot rand\cdot \left(Xbest_{j}-subPopK_{j}^{i}\right)\\ +c_{3}\cdot rand\cdot \left(pbest-subPopK_{j}^{i}\right)+c_{4}\cdot rand\cdot \left(gbest-subPopK_{j}^{i}\right) \end{array}$$

In the equations, $c_{1}$ represents the inertia factor, while $c_{2},\;{\;c}_{3}$, and $c_{4}$ are learning factors. The variable *rand* refers to a uniformly distributed random number between 0 and 1. $V_{j}^{i}$ denotes the velocity of an individual in the population that migrates to another subpopulation, while ${Xbest}_{j}$ represents the optimal historical position of an individual in the population. The pbest is the optimal historical solution among individuals in a subpopulation, while gbest is the global optimal solution among all subpopulations. The $sub{PopK}_{j}^{i}$ represents an individual within a particular subpopulation, where *i* denotes the generation, and *j* denotes the index of the individual within the subpopulation.

In addition, Equation (8) is used to limit the velocity between *Vmin* and *Vmax*, ensuring the stability and accuracy of the search process. *Vmin* and *Vmax* represent the minimum and maximum values of the velocity, respectively.

(8) $$V_{j}^{i+1}=Clip\left(V_{j}^{i+1},Vmin,Vmax\right)\;$$

#### 2.2.3. Adaptive Reconstruction and Elimination Mechanism

After completing the search process, we employed the K-means clustering method to restructure all the subpopulations. We selected the optimal subpopulation solutions *pbest1*, *pbest2*, …, *pbestK* from each subpopulation *subPop1*, *subPop2*, …, *subPopK* as the cluster centers. Then, we calculated the Euclidean distance ${\left\|{Pop}_{i}-pbestK\right\|}_{2}$ between all the individuals ${Pop}_{i}$ and optimal solutions of each subpopulation *pbest1*, *pbest2…*, *pbestK*. Finally, each individual ${Pop}_{i}$ was assigned to the subpopulation (*subPop1*, *subPop2*…, *subPopK*) with the closest distance to its corresponding cluster center. As shown in Algorithm 2.

**Figure 4:**
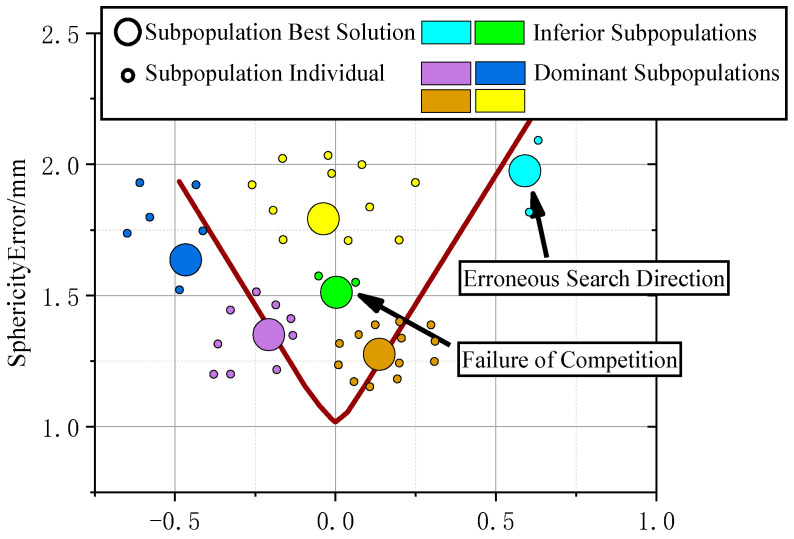
Schematic diagram of the reasons for the formation of inferior subpopulations. Each color in the diagram represents a subpopulation (it should be noted that Figure 4 is not obtained from the actual experiment but is a simulation presentation using a two-dimensional view to explain more clearly the two reasons that lead to the creation of inferior subpopulations).

After recombining the subpopulations using clustering methods, each subpopulation is distributed into separate local spaces within the search space ***R*** without overlapping. Any subpopulations that do not acquire adequate individuals following reclustering are classified as inferior. Typically, population individuals converge toward the ideal solution. However, two situations can lead to the production of inferior subpopulations: one is the erroneous search direction, and the other is the failure to compete with other subpopulations, even though the search direction of this subpopulation is correct. Figure 4 is a two-dimensional schematic diagram that shows the reasons for producing inferior subpopulations after the recombination of subpopulations. Each color represents a different subpopulation. 

To address this issue, we have developed an elimination mechanism to remove subpopulations that cannot cluster enough individuals. If a reclustered subpopulation’s number of individuals falls below the threshold, it is considered inferior and will be eliminated. The search stops after a certain proportion of inferior subpopulations have been eliminated. By using an adaptive subpopulation set, the global optimum can be found efficiently and accurately.
**Algorithm 2: Adaptive Reconstruction and Elimination Mechanism****Input:**-Search space ***R***-Population **Pop**-Subpopulations *subPop1*, *subPop2*, *…*, *subPopK*-Optimal solutions of subpopulations *pbest1*, *pbest2*, *…*, *pbestK***Output:**-The global optimal solution *gbest*
1: **While** the number of subpopulations *K* falls below the threshold: 2:  Perform the search process (as described in Section 2.2.2); 3:  Utilize the *K*-means clustering method to reorganize all subpopulations; 4:  Select *pbest*1, *pbest*2, *…*, *pbestK* as the cluster centers from each *subPop1*, *subPop2*, *…*, *subPopK*; 5:  $\mathrm{for}\;\mathrm{each}\;\mathrm{individual}\;{Pop}_{i}$ in **Pop**: 6:    $\mathrm{Calculate}\;\mathrm{Euclidean}\;\mathrm{distance}\;{\left\|{Pop}_{i}-pbestK\right\|}_{2}$$\;\mathrm{between}\;{Pop}_{i}$ and each *pbest1*, *pbest2*, *…*, *pbestK*; 7:    $\mathrm{Assign}\;{Pop}_{i}$ to the subpopulation with the closest distance to its corresponding cluster center; 8:  **end for** 9:  **for** each *subPopK*: 10:    **if** the number of individuals in a subpopulation is less than the Threshold: 11:      Eliminate the subpopulation *subPopK*; 12:    **end if** 13:  **end for** 14:  Continue the search process; 15: *end while* 16: **Return** the global optimal solution *gbest*.

## 3. Results

### 3.1. Simulated Experiments

#### 3.1.1. The First Part of the Simulation Experiments

Three groups of simulation datasets were generated to verify algorithm accuracy in different manufacturing processes for the HSRs with known sphericity errors. Table 1 presents each simulation dataset’s sphericity errors, radii, and number of simulated samples. 

The sphericity error in the simulation data was controlled using the following method [42]. The points located on the surface of the concentric sphere are referred to as control points. Based on the coefficient equation of the sphere in polar coordinates, as shown in Equation (9), ten control points were randomly generated on the inner and outer surfaces of a concentric sphere with radii of *R* and *R + S*_0_, respectively. Thus, three groups of simulation sampling coordinates $P^{i},i=1,2,3,\ldots ,12$ are generated. The 20 control points can constrain the sphericity error of the simulation datasets for the difference $S_{0}$ between the radii of the inner and outer spheres. This approach provided reliable data to verify the algorithm’s accuracy under different manufacturing processes.

(9) $$X=R\cdot \cos\left(\theta \right)\cdot \sin\left(\phi \right),\;Y=R\cdot \sin\left(\theta \right)\cdot \cos\left(\phi \right),\;Z=R\cdot \cos\left(\phi \right)$$

The initial total number of populations was set to 300, and the initial number *K* of subpopulations was 10. The subpopulation was eliminated when the number of individuals in the subpopulation was less than 3. The threshold of population elimination number was 3, and the search was finished when the number of subpopulations was less than 3. Then, we could calculate the above simulation data 10 times independently and calculate the mean and standard deviation of the results for each dataset.

**Figure 5:**
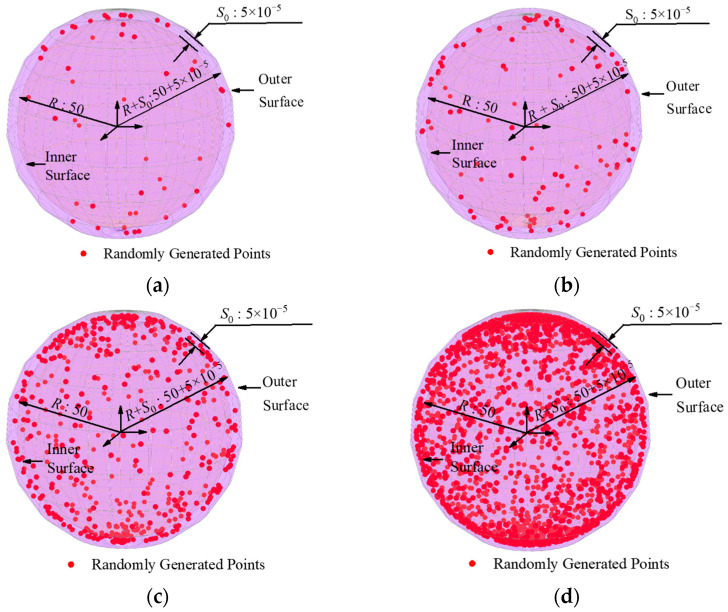
Schematic diagram of the simulation data distribution in group A (unit: mm): (**a**) 50 sampling points; (**b**) 100 sampling points; (**c**) 500 sampling points; (**d**) 2000 sampling points.

The datasets within group A had identical sphericity error values of 0.00005 mm and a radius of 50 mm, with varying numbers of sampling points at 50, 100, 500, and 2000, respectively. Figure 5 shows the distribution of group A’s dataset on the sphere surface. These datasets were processed using the least squares method and our algorithm, and the resulting analysis is presented in Table 2.

**Figure 6:**
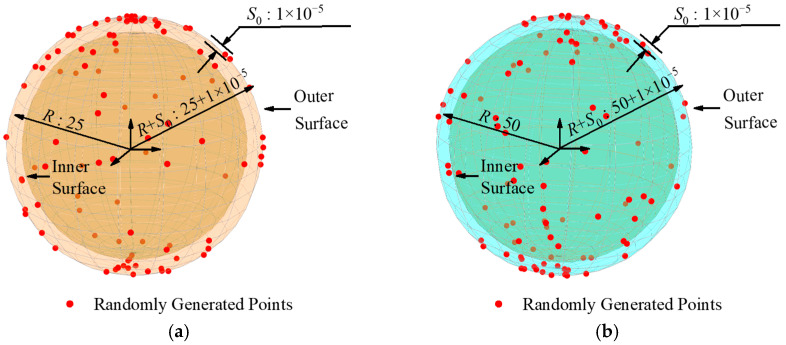

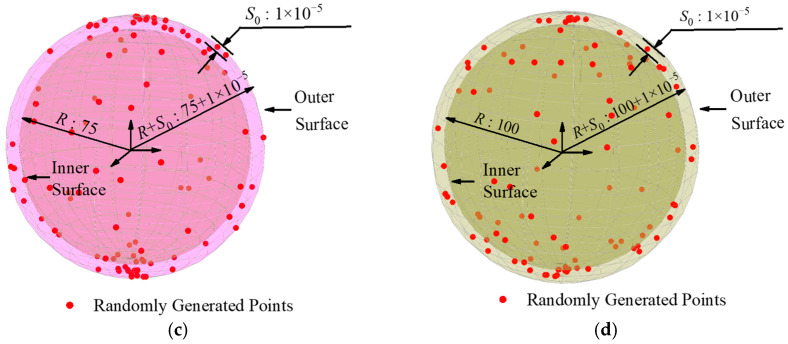
Schematic diagram of the simulation data distribution in group B (unit: mm). (**a**) Radius: 25. (**b**) Radius: 50. (**c**) Radius: 75. (**d**) Radius: 100.

The datasets in group B had the same sphericity error of 0.00001 mm and the sampling point number of 100. Their radii were 25 mm, 50 mm, 75 mm, and 100 mm, respectively. The distribution of the group B dataset on the sphere surface is shown in Figure 6. The data of group B were processed using the least squares method and our algorithm, and the processing results are shown in Table 3.

**Figure 7:**
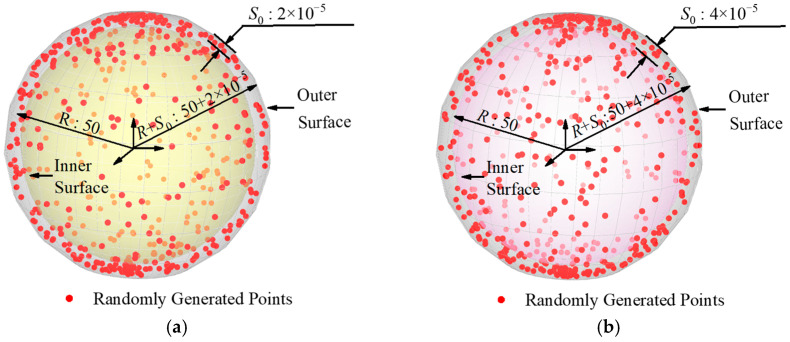

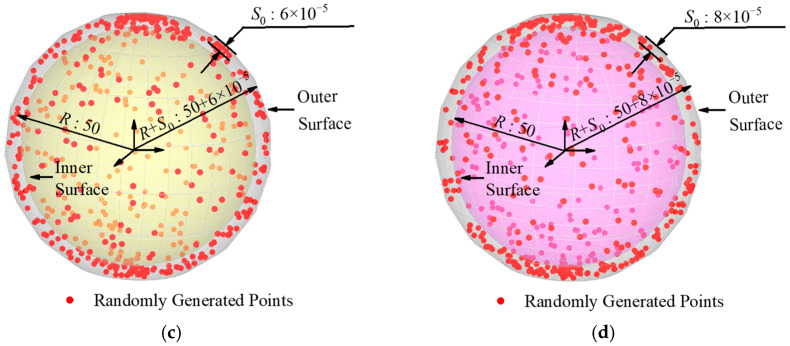
Schematic diagram of the simulation data distribution in group C. (Unit: mm). (**a**) Sphericity Error: 0.00002. (**b**) Sphericity Error: 0.00004. (**c**) Sphericity Error: 0.00006. (**d**) Sphericity Error: 0.00008.

The datasets in group C had the same radius of 50 mm and the sampling point number of 500. Their sphericity errors were 0.00002 mm, 0.00004 mm, 0.00006 mm, and 0.00008 mm, respectively. Figure 7 shows the distribution of group C’s simulation dataset on the sphere surface. The data of group C were processed using the least squares method and our algorithm, and the processing results are shown in Table 4.

From the above experimental results, it can be seen that our algorithm can accurately evaluate the spherical sphericity error when dealing with a simulation dataset of different sphericity errors, different radii, and different sampling points. Compared with the least squares method, our algorithm has about 10% accuracy improvement, and the standard deviation of the algorithm solution is in the order of 10^−9^ mm.

#### 3.1.2. The Second Part of the Simulation Experiments

Additionally, to assess the robustness and evaluation accuracy of the algorithm, we generated six groups of simulated data with known sphericity errors using the aforementioned method from the first part of the simulation experiment. 

The difference in each group of simulated data lies in the number of sampling points, with each simulating 50, 100, 500, 1000, 1500, and 2000 sampling points, respectively. Each group of simulated data contains 100 spheres with different radii and ten types of sphericity errors. The radii of the simulated data are uniformly distributed in the range of [1 mm, 100 mm]. The sphericity errors are uniformly distributed in the range of [1 × 10^−5^ mm, 1 × 10^−4^ mm]. Each group consists of 1000 datasets, for a total of 6000 datasets.

We conducted simulation experiments based on these six groups, a total of 6000 datasets. And we performed statistical analysis on the deviation value *D* between the experimentally obtained sphericity error result $S_{E}$ and the ideal sphericity error value $S_{I}$. We used Formula (10) to calculate the deviation value.

(10) $$D=S_{E}-S_{I}$$

The results, presented in Table 5, show that each group’s experimental results $S_{E}$ have a mean deviation from the ideal sphericity error $S_{I}$ of approximately 10^−9^ mm. It is noticeable that the number of sampling points has a negligible effect on the experimental results, indicating the algorithm’s capability to access the sphericity error effectively under varying process requirements. The standard deviation of *D* for each group’s experiment results is also approximately 10^−9^ mm, which demonstrates the algorithm’s high level of robustness. And the time required for each sphericity error assessment is less than 1 s. Extensive experiments have provided evidence that the algorithm can effectively satisfy the requirements of precision, speed, and robustness for sphericity error assessment in the manufacturing process of the HSRs. This suggests that there is potential for high-precision sphericity error assessment in the manufacturing process of the HSRs.

### 3.2. Comparison Experiments

In addition, to further verify the accuracy of the algorithm, we conducted experiments using the dataset provided by the authoritative literature in the field of sphericity error research. We then compared our results with the works in the literature that cite the same dataset. The three datasets include the surface sampling data of the hemispherical part and the simulation data generated on two concentric spheres. We then performed the experiments ten times independently based on each dataset and calculated the mean and standard deviation of the experimental results.

**Figure 8:**
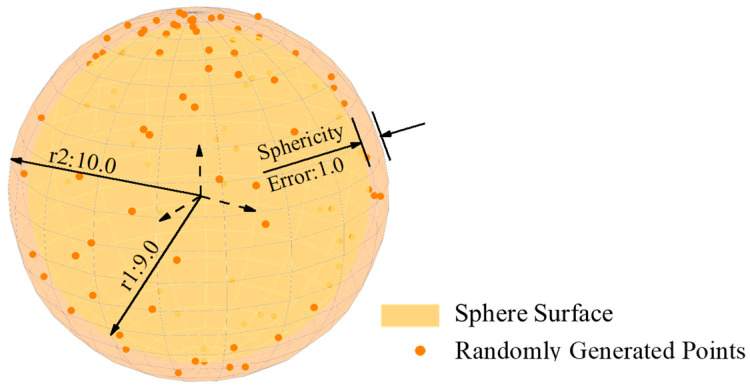
Data distribution of dataset 1 (unit: mm).

Dataset 1 is given by reference [42], consisting of 100 coordinate points with a sphericity error of 1.0 mm. The sphericity error of this dataset has been verified by several works [34,42] and the data distribution is shown in Figure 8. Ten independent experiments were conducted based on dataset 1. The mean value of sphericity error in the experimental results was 1.00000000035 mm, and the standard deviation of the results was 2.4 × 10^−10^ mm. The detailed experimental results are presented in Table A1 in Appendix A, while Table 6 shows a comparison with existing literature results.

**Figure 9:**
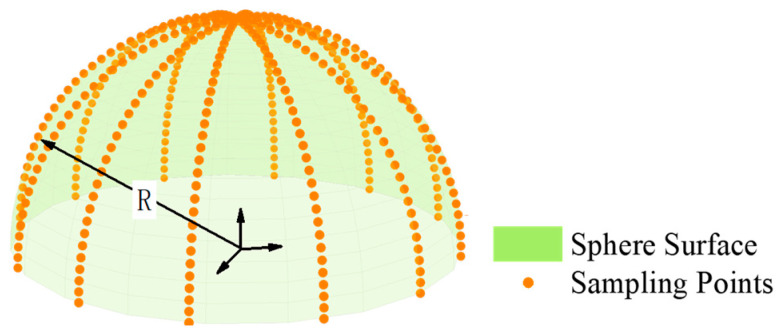
Schematic diagram of the distribution of dataset 2.

Dataset 2 is given by the reference [45] and consists of 384 coordinate points. All 384 coordinate points were obtained from real spherical sampling using the birdcage method, and were evenly distributed on 12 lines on the sphere. The distribution of dataset 2 is shown in Figure 9. For dataset 2, ten groups of replicate experiments were each performed. The results of the ten experiments are shown in Table A2 in Appendix A; the mean experimental result is 0.015384870588 mm, with a standard deviation of 6.4 × 10^−11^ mm. 

A comparison with the results in the literature is shown in Table 7. Compared with the results in reference [45], although both algorithms gave the same results after rounding to the same significant figures, our algorithm provided more significant figures. It exhibited a higher uncertainty, indicating that the present algorithm will have a significant advantage in the mass production of HSRs.

**Figure 10:**
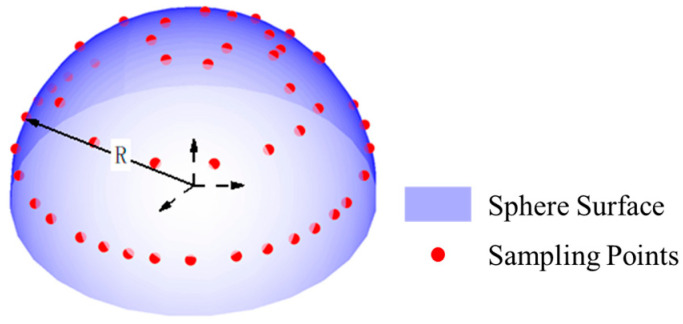
Schematic diagram of the distribution of dataset 3.

Dataset 3 is given in reference [13], which is sampled from the ultra-precision quartz hemisphere manufactured by Taylor Hobson, and it contains 50 coordinate points. The distribution of the coordinate sampling points is shown in Figure 10. For dataset 3, ten groups of replicate experiments were each performed. The results of the ten experiments are shown in Table A3 in Appendix A; the mean experimental result is 0.00087023058 mm, with a standard deviation of 0.1 × 10^−10^ mm. 

A comparison with the results in the literature is shown in Table 8. The accuracy of the present algorithm is improved by about 15 nanometers compared to the results of reference [13]. Given the stringent geometric and positional tolerances required for HSRs, their measurement accuracy needs to reach the nanometer level. In this high-precision measurement scenario, the accuracy improvement of the algorithm in this paper is highly significant.

### 3.3. Practical Application Experiments

**Figure 11:**
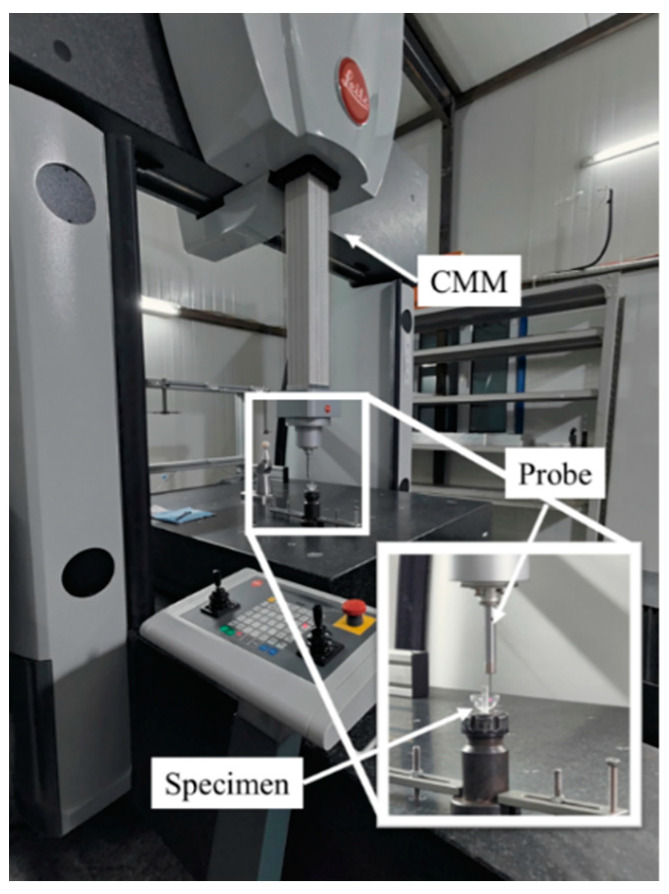
Schematic diagram of the experimental equipment.

Finally, we applied the algorithm to sphericity error detection in the manufacturing process of the HSRs. In a controlled environment, we used a Hexagon coordinate measuring machine to measure the inner and outer contours of the thin-walled spherical shell of the HSR sample. The sampling diagram is shown in Figure 11, while the corresponding data for the inner and outer contours are given in the Table A6 and Table A7 in Appendix A.

**Figure 12:**
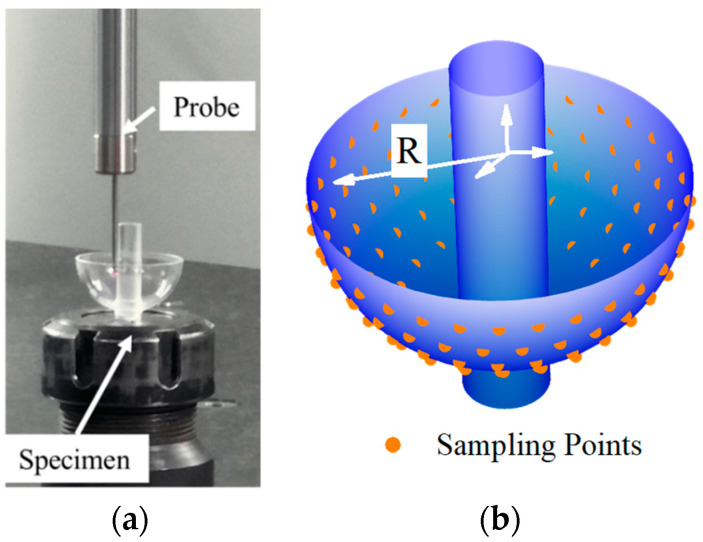
Schematic diagram of sampling and data reconstruction of the HSR’s inner contour. (**a**) Actual image of the HSR during measurement. (**b**) The schematic illustrates the distribution of measurement points on the surface of the HSR.

The internal contour sampling is shown in Figure 12a. We fixed the internal contour of the resonator upward on the measurement platform and obtained 130 coordinate data by measuring five cross-sections. By reconstructing these internal contour data (see Figure 12b), we conducted ten repeated experiments on the internal contour sampling data using the same algorithm parameters aforementioned. The results are shown in Table A4 in Appendix A.

The average value of the ten experiments was 0.002413104397 mm, with a standard deviation of 6.0 × 10^−11^ mm. The sphericity error data provided by the CMM was 0.0029 mm, as shown in Table 9. Based on the experimental results of the internal contour sampling data, the algorithm achieved approximately 17% higher precision compared to the sphericity error data provided by the CMM.

**Figure 13:**
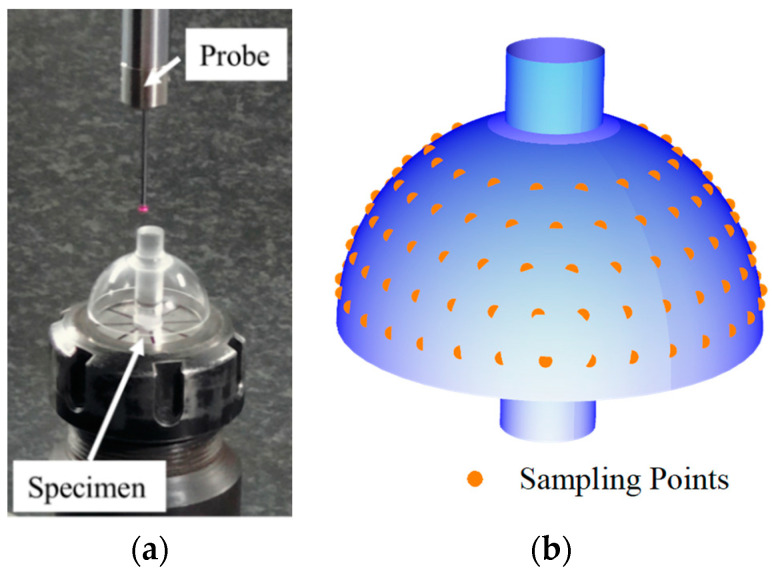
Schematic diagram of sampling and data reconstruction of the hemispherical shell resonator outer contour (**a**) Actual image of the HSR during measurement. (**b**) The schematic illustrates the distribution of measurement points on the surface of the HSR.

In terms of outer contour sampling, we employed a method similar to the one used for inner contour, as shown in Figure 13. We measured five sections on the outer contour, obtaining 130 coordinate data, and reconstructed the outer contour data accordingly.

Ten repeated experiments were conducted on the sampled outer contour data, and the results are shown in Table A5 in Appendix A. The average value of the ten experiments is 0.002799199921 mm, with a standard deviation of 2.1 × 10^−11^ mm. The CMM provided a sphericity error evaluation of 0.0029 mm. And the algorithm’s accuracy improved by approximately 4% over the CMM result in the experiment based on the outer contour data, as shown in Table 10. 

## 4. Discussion

The sphericity error of the HSR’s thin-walled spherical shell is a crucial shape error affecting the performance of the HRG. The sphericity error assessment method used in the manufacturing process of the HSRs must have high speed, high accuracy, and high robustness when handling large datasets. To meet the requirements of this specific scenario, we particularly focused on the distribution characteristics of sphericity errors near the ideal center of the sphere. Near the ideal center of the sphere, the change in sphericity errors was complex. In contrast, far from the ideal center, the gradient of sphericity errors changed significantly, showing a rapid decrease trend. We have designed an adaptive multi-population sphericity error assessment method based on this characteristic. In the initial stages of the search, this method utilizes global and subpopulation optimal solutions to guide the subpopulations to converge on regions closer to the ideal sphericity error. In the later stages of the search, inferior subpopulations are eliminated, and individuals from the inferior subpopulations converge into the remaining subpopulations, performing detailed searches near the ideal solution. The search stops after eliminating a certain number of subpopulations.

In simulation experiments, we have demonstrated the algorithm’s ability to maintain high accuracy and robustness in different machining process scenarios. When processing simulation datasets with different sphericity errors, radii, and numbers of sampling points, the algorithm ensures an evaluation accuracy on the order of 10^−9^ mm. Moreover, when processing up to 6000 simulation datasets, the deviation between the algorithm’s solution and the ideal sphericity error remains at approximately −3 × 10^−9^ mm, demonstrating the algorithm’s high precision and robustness. This confirms that the algorithm can meet the requirements for sphericity error assessment in the manufacturing process of the HSRs when handling different spherical contour sampling data.

In addition, based on the datasets provided by the authoritative literature in the field of sphericity error research, we conducted comparison experiments with the literature results that cited the same authoritative datasets. The comparative results also indicate that our algorithm has a significant advantage in the precision of sphericity error assessment. Finally, we applied the algorithm to the sphericity error assessment in the manufacturing process of the HSRs. When measuring hemispherical shell resonator samples, our algorithm improved the sphericity error assessment accuracy of the inner and outer contour sampling data by 17% and 4%, respectively, compared with the results from a CMM. 

In the field of high-precision error measurement and evaluation, strict control of the error in each step, from the measurement equipment to the evaluation algorithm, is essential to ensure high-precision error measurement and evaluation. The results of practical application experiments demonstrate that our algorithm improves the measurement accuracy of sphericity error of the inner and outer spherical shells by about 480 nm and 110 nm, respectively. The hundred-nanometer level of accuracy improvement achieved by the algorithm improvement is significant, and has significant economic value.

The above results demonstrate the effective application of the algorithm in the specific scenario of sphericity error assessment for the HSRs. We believe that this method has the potential to be widely applied in future research and engineering practice. In the future, we will integrate this approach into a piece of form error measurement equipment to provide reliable measurement and evaluation accuracy for the manufacture of HSRs, thus effectively improving the performance of HRGs.

## Figures and Tables

**Table 1:** Simulation datasets. (Units: mm).

Group	No.	Sphericity Error	Radius	Number of Simulation Points
A	(a)	0.00005	50	50
	(b)	0.00005	50	100
	(c)	0.00005	50	500
	(d)	0.00005	50	2000
B	(a)	0.00001	25	100
	(b)	0.00001	50	100
	(c)	0.00001	75	100
	(d)	0.00001	100	100
C	(a)	0.00002	50	500
	(b)	0.00004	50	500
	(c)	0.00006	50	500
	(d)	0.00008	50	500

**Table 2:** Sphericity error evaluation results of group A data. (Unit: mm).

No.	Simulation Data True Value	Mean Value (Our Algorithm)	Standard Deviation (Our Algorithm)
(a)	0.00005	0.0000500037	1.8 × 10^−9^
(b)	0.00005	0.0000500059	3.7 × 10^−9^
(c)	0.00005	0.0000500048	1.8 × 10^−9^
(d)	0.00005	0.0000500057	1.9 × 10^−9^

**Table 3:** Sphericity error evaluation results of group B data (unit: mm).

No.	Simulation Data True Value	Mean Value (Our Algorithm)	Standard Deviation (Our Algorithm)
(a)	0.00001	0.0000100019	1.1 × 10^−9^
(b)	0.00001	0.0000100018	7.0 × 10^−9^
(c)	0.00001	0.0000100021	1.1 × 10^−9^
(d)	0.00001	0.0000100031	2.4 × 10^−9^

**Table 4:** Sphericity error evaluation results of group C data. (Unit: mm).

No.	Simulation Data True Value	Mean Value (Our Algorithm)	Standard Deviation (Our Algorithm)
(a)	0.00002	0.0000200026	2.1 × 10^−9^
(b)	0.00004	0.0000400054	2.7 × 10^−9^
(c)	0.00006	0.0000600049	4.4 × 10^−9^
(d)	0.00008	0.0000800060	2.8 × 10^−9^

**Table 5:** Statistical results of deviations between experimental results and ideal values for each group of datasets (unit: mm).

Group	1	2	3	4	5	6
Number of simulation points	50	100	500	1000	1500	2000
Mean deviation *D* (×10^−9^)	−3.45	−3.48	−3.46	−3.53	−3.46	−3.58
Standard deviation *D* (×10^−9^)	2.34	2.46	2.38	2.49	2.40	2.39
Median deviation *D* (×10^−9^)	−2.86	−2.93	−2.92	−3.02	−2.96	−3.06

**Table 6:** Comparison with results based on dataset 1. (Rounding is as given in the respective papers; units: mm).

Algorithm	Spherical Center Coordinate (*X*, *Y*, *Z*)	Sphericity Error
Least squares	(−0.01913, 0.08935, 0.01762)	1.15224
[34]	(0.00000000, 0.00000000, 0.00000000)	1.00000000
[42]	(0.00000, 0.00000, 0.00000)	1.00000
Ours	(0.000000002, 0.000000000, 0.000000000)	1.00000000035

The number of zeros after the decimal points represents the precision provided in the original data from the cited references.

**Table 7:** Comparison with results based on dataset 2. (Rounding is as given in the respective papers; units: mm).

Algorithm	Spherical Center Coordinate (*X*, *Y*, *Z*)	Sphericity Error
Least squares	(−0.0001146, −0.0002871, 0.0078932)	0.01639862
[13]	(−0.0011426, 0.0000027, 0.0107779)	0.0154077
[45]	(0.000179, −0.000332, 0.011747)	0.015385
Ours	(0.000212598362, −0.000350698709, 0.011748193020)	0.015384870588

**Table 8:** Comparison with results based on dataset 3. (Rounding is as given in the respective papers; units: mm).

Algorithm	Spherical Center Coordinate (*X*, *Y*, *Z*)	Sphericity Error
Least squares	(−0.000154, 0.000057, 0.000611)	0.001061
[13]	(0.00004026, 0.00009520, 0.00042457)	0.00088537
Ours	(−0.00004684948, 0.0001022795, 0.00066651430)	0.00087023058

**Table 9:** Comparison of experimental results based on the inner contour sampling data (unit: mm).

Algorithm	Spherical Center Coordinate (*X*, *Y*, *Z*)	Sphericity Error
CMM	—	0.0029
Ours	(−0.000585909974, −0.001064711697, −9.845127859718)	0.002413104397

**Table 10:** Comparison of experimental results based on the outer contour sampling data (unit: mm).

Algorithm	Spherical Center Coordinate (*X*, *Y*, *Z*)	Sphericity Error
CMM	—	0.0029
Ours	(0.009162260968, 0.013219540972, −22.120848936292)	0.002799199921

## Data Availability

All data that support the findings of this study are included within this article.

## Appendix A

**Table A1:** Results of ten experiments based on dataset 1 (unit: mm).

Experiment	1	2	3	4
Sphericity Error	1.00000000076	1.00000000000	1.00000000000	1.00000000049
Experiment	5	6	7	8
Sphericity Error	1.00000000010	1.00000000057	1.00000000057	1.00000000019
Experiment	9	10	Mean Value	Standard Deviation
Sphericity Error	1.00000000049	1.00000000034	1.00000000035	2.4 × 10^−10^

**Table A2:** Results of ten experiments based on dataset 2 (unit: mm).

Experiment	1	2	3	4
Sphericity Error	0.015384870552	0.015384870554	0.015384870559	0.015384870551
Experiment	5	6	7	8
Sphericity Error	0.015384870743	0.015384870680	0.015384870586	0.015384870552
Experiment	9	10	Mean Value	Standard Deviation
Sphericity Error	0.015384870553	0.015384870551	0.015384870588	6.4 × 10^−11^

**Table A3:** Results of ten experiments based on dataset 3 (unit: mm).

Experiment	1	2	3	4
Sphericity Error	0.00087023059	0.00087023059	0.00087023057	0.00087023057
Experiment	5	6	7	8
Sphericity Error	0.00087023062	0.00087023057	0.00087023057	0.00087023057
Experiment	9	10	Mean Value	Standard Deviation
Sphericity Error	0.00087023058	0.00087023059	0.00087023058	0.1 × 10^−10^

**Table A4:** Results of ten experiments based on the inner contour sampling data (unit: mm).

Experiment	1	2	3	4
Sphericity Error	0.002413104367	0.002413104367	0.002413104396	0.002413104574
Experiment	5	6	7	8
Sphericity Error	0.002413104369	0.002413104386	0.002413104372	0.002413104365
Experiment	9	10	Mean Value	Standard Deviation
Sphericity Error	0.002413104364	0.002413104393	0.002413104397	6.0 × 10^−11^

**Table A5:** Results of ten experiments based on the outer contour sampling data (unit: mm).

Experiment	1	2	3	4
Sphericity Error	0.002799199953	0.002799199924	0.002799199909	0.002799199909
Experiment	5	6	7	8
Sphericity Error	0.002799199964	0.002799199903	0.002799199897	0.002799199937
Experiment	9	10	Mean Value	Standard Deviation
Sphericity Error	0.002799199910	0.002799199902	0.002799199921	2.1 × 10^−11^

**Table A6:** Original data sampling from the HSR’s inner contour (unit: mm).

No.	X	Y	Z	No.	X	Y	Z
1	14.06391	−2.79998	−12.5351	66	7.300748	−9.80718	−17.8104
2	13.24758	−5.48966	−12.5351	67	4.84431	−11.2265	−17.8096
3	11.92215	−7.96828	−12.5351	68	2.124613	−12.0415	−17.8093
4	10.14031	−10.1405	−12.5343	69	−0.71186	−12.2078	−17.808
5	7.96813	−11.9237	−12.5347	70	−3.50812	−11.7146	−17.8087
6	5.48934	−13.249	−12.5348	71	−6.11441	−10.5902	−17.8087
7	2.799466	−14.0654	−12.5345	72	−8.39038	−8.89471	−17.8103
8	−0.00029	−14.3413	−12.5351	73	−10.2152	−6.71947	−17.8099
9	−2.79967	−14.0658	−12.5349	74	−11.4896	−4.18244	−17.8103
10	−5.48932	−13.25	−12.5346	75	−12.1447	−1.42013	−17.8096
11	−7.96804	−11.9251	−12.5351	76	−12.1431	1.419142	−17.812
12	−10.1407	−10.1421	−12.5353	77	−11.49	4.179747	−17.81
13	−11.9241	−7.96898	−12.5354	78	−10.2141	6.717131	−17.8121
14	−13.2495	−5.48954	−12.5354	79	−8.39022	8.891081	−17.8118
15	−14.0659	−2.79858	−12.5353	80	−6.114	10.58636	−17.8111
16	−14.3417	0.00106	−12.5359	81	−3.5074	11.71077	−17.8112
17	−14.0661	2.796336	−12.5354	82	−0.7108	12.20431	−17.8102
18	−13.2497	5.48733	−12.536	83	2.125983	12.03835	−17.8106
19	−11.9249	7.96601	−12.5358	84	4.84612	11.22314	−17.8105
20	−10.1414	10.13943	−12.5358	85	7.302828	9.803704	−17.8104
21	−7.96936	11.92059	−12.5345	86	9.366855	7.855192	−17.8105
22	−5.48952	13.24811	−12.5363	87	10.92633	5.483563	−17.8098
23	−2.79955	14.06385	−12.5374	88	11.89697	2.815969	−17.809
24	0.00163	14.33978	−12.5363	89	12.22547	−0.0009	−17.8097
25	2.801406	14.06353	−12.5365	90	10.44835	−0.00072	−20.0291
26	5.49191	13.24623	−12.5367	91	10.06086	2.816411	−20.0296
27	7.97046	11.92077	−12.5358	92	8.927452	5.425589	−20.0302
28	10.14232	10.13717	−12.5362	93	7.132952	7.63352	−20.0296
29	11.92519	7.9638	−12.5355	94	4.809464	9.274656	−20.0295
30	13.24949	5.48477	−12.5357	95	2.127819	10.22867	−20.0293
31	14.06516	2.794226	−12.5351	96	−0.71363	10.42328	−20.0295
32	14.34012	−0.0007	−12.5352	97	−3.50091	9.843635	−20.03
33	13.54089	−0.00064	−15.2801	98	−6.02698	8.53451	−20.03
34	13.24589	2.811995	−15.2794	99	−8.10515	6.591765	−20.0312
35	12.37083	5.505222	−15.2808	100	−9.58438	4.160251	−20.0304
36	10.95676	7.95639	−15.2798	101	−10.3514	1.420982	−20.0309
37	9.062855	10.06066	−15.2802	102	−10.351	−1.42519	−20.0309
38	6.773564	11.72446	−15.2813	103	−9.5825	−4.16489	−20.0314
39	4.187366	12.87699	−15.2807	104	−8.1052	−6.59663	−20.0299
40	1.417191	13.46638	−15.2808	105	−6.02506	−8.53726	−20.031
41	−1.41645	13.4667	−15.2806	106	−3.49968	−9.84611	−20.0307
42	−4.18607	12.87796	−15.2805	107	−0.71323	−10.4248	−20.0306
43	−6.77237	11.72521	−15.2818	108	2.127999	−10.2308	−20.0296
44	−9.0629	10.06155	−15.2807	109	4.809164	−9.27709	−20.0297
45	−10.9573	7.95728	−15.2803	110	7.132122	−7.63599	−20.03
46	−12.372	5.505872	−15.2808	111	8.926642	−5.42945	−20.0299
47	−13.2467	2.812985	−15.28	112	10.06015	−2.81986	−20.0298
48	−13.5429	−0.00134	−15.2798	113	7.780166	−2.83326	−21.8577
49	−13.2464	−2.81737	−15.2804	114	6.342835	−5.32246	−21.858
50	−12.3715	−5.51021	−15.2797	115	4.140738	−7.17079	−21.8582
51	−10.956	−7.96148	−15.2803	116	1.438711	−8.1555	−21.8579
52	−9.06162	−10.0654	−15.2797	117	−1.43887	−8.15517	−21.8585
53	−6.77156	−11.7291	−15.2798	118	−4.14125	−7.17202	−21.8582
54	−4.186	−12.8807	−15.2788	119	−6.34357	−5.32343	−21.8584
55	−1.41632	−13.4691	−15.2792	120	−7.78083	−2.83298	−21.8587
56	1.416831	−13.4683	−15.2802	121	−8.27767	−0.00058	−21.8605
57	4.187006	−12.8795	−15.279	122	−7.78016	2.829985	−21.8592
58	6.771604	−11.7266	−15.2817	123	−6.34208	5.320018	−21.8594
59	9.061275	−10.063	−15.2795	124	−4.1395	7.166962	−21.8598
60	10.95539	−7.95932	−15.2801	125	−1.43796	8.151543	−21.8589
61	12.37066	−5.50804	−15.279	126	1.439871	8.151423	−21.8585
62	13.2455	−2.81595	−15.2787	127	4.142618	7.167132	−21.8581
63	11.89603	−2.82017	−17.8091	128	6.344425	5.318658	−21.8578
64	10.9247	−5.4873	−17.8102	129	7.780806	2.828805	−21.858
65	9.366585	−7.85914	−17.8085	130	8.280216	−0.00115	−21.8574

**Table A7:** Original data sampling from the HSR’s outer contour (unit: mm).

No.	X	Y	Z	No.	X	Y	Z
1	8.269614	−3.01208	−9.46766	66	11.58246	5.813837	−13.7638
2	6.739105	−5.65809	−9.47043	67	12.61006	2.985381	−13.765
3	4.396742	−7.62109	−9.47324	68	12.95667	−0.00137	−13.7666
4	1.5259	−8.66367	−9.47553	69	14.33326	−0.00126	−16.4409
5	−1.52552	−8.66241	−9.47665	70	14.02288	2.977565	−16.4396
6	−4.3944	−7.61834	−9.47707	71	13.09855	5.828858	−16.4385
7	−6.73461	−5.65534	−9.4761	72	11.60075	8.42625	−16.4384
8	−8.26352	−3.01037	−9.47485	73	9.594365	10.6552	−16.4374
9	−8.79604	0.00048	−9.47293	74	7.168796	12.41768	−16.4382
10	−8.26717	3.007485	−9.47006	75	4.429074	13.63624	−16.4376
11	−6.74022	5.655312	−9.4672	76	1.497249	14.25827	−16.4385
12	−4.39891	7.622298	−9.46508	77	−1.49721	14.2568	−16.4393
13	−1.52677	8.668757	−9.46315	78	−4.42728	13.63181	−16.4396
14	1.52802	8.669507	−9.46182	79	−7.16454	12.41141	−16.4418
15	4.401722	7.623758	−9.4611	80	−9.58773	10.64795	−16.443
16	6.744675	5.657292	−9.46136	81	−11.5909	8.41875	−16.4438
17	8.273184	3.007495	−9.46279	82	−13.0858	5.822578	−16.4442
18	8.802954	−0.00149	−9.46567	83	−14.0076	2.973985	−16.4452
19	11.08956	−0.00117	−11.4121	84	−14.317	−0.00067	−16.446
20	10.68025	2.989809	−11.41	85	−14.0013	−2.98	−16.4473
21	9.477348	5.762251	−11.4084	86	−13.0744	−5.82473	−16.4484
22	7.572208	8.10625	−11.4088	87	−11.5761	−8.41537	−16.4487
23	5.102576	9.849474	−11.4088	88	−9.57252	−10.6382	−16.4494
24	2.255321	10.86076	−11.4094	89	−7.15134	−12.3964	−16.4508
25	−0.7565	11.06518	−11.41	90	−4.41807	−13.6121	−16.4489
26	−3.71192	10.44851	−11.4104	91	−1.49343	−14.2344	−16.4493
27	−6.3925	9.05727	−11.4119	92	1.495329	−14.236	−16.4488
28	−8.59862	6.994155	−11.414	93	4.421844	−13.6162	−16.4474
29	−10.1661	4.413339	−11.417	94	7.157076	−12.4019	−16.4463
30	−10.9781	1.505938	−11.4196	95	9.581115	−10.6449	−16.4447
31	−10.9749	−1.51204	−11.4209	96	11.58775	−8.4217	−16.4436
32	−10.1582	−4.41669	−11.4221	97	13.0887	−5.82926	−16.4429
33	−8.58983	−6.99375	−11.4241	98	14.01724	−2.98174	−16.4411
34	−6.38363	−9.05201	−11.4246	99	14.87208	−2.95921	−19.3345
35	−3.70554	−10.439	−11.4248	100	14.00591	−5.80333	−19.335
36	−0.75458	−11.0539	−11.4256	101	12.60221	−8.42183	−19.3354
37	2.252461	−10.8487	−11.4233	102	10.71395	−10.7169	−19.3359
38	5.096076	−9.84021	−11.4212	103	8.4147	−12.599	−19.3363
39	7.563688	−8.10186	−11.4192	104	5.79394	−13.9958	−19.3368
40	9.470028	−5.76202	−11.4159	105	2.951894	−14.8547	−19.3372
41	10.6758	−2.99348	−11.4138	106	0.00024	−15.1431	−19.3376
42	12.60656	−2.98892	−13.7703	107	−2.95078	−14.8513	−19.3374
43	11.57333	−5.81496	−13.77	108	−5.7902	−13.9898	−19.3376
44	9.917995	−8.32585	−13.772	109	−8.40791	−12.5912	−19.3378
45	7.729232	−10.3867	−13.7744	110	−10.7039	−10.7084	−19.3378
46	5.12401	−11.8869	−13.7755	111	−12.5886	−8.4144	−19.3376
47	2.245547	−12.7462	−13.7772	112	−13.99	−5.79676	−19.337
48	−0.75141	−12.9199	−13.7796	113	−14.854	−2.95611	−19.3369
49	−3.70747	−12.3973	−13.7789	114	−15.1478	−0.00059	−19.3366
50	−6.46593	−11.2077	−13.779	115	−14.8601	2.953244	−19.336
51	−8.87696	−9.4143	−13.7791	116	−14.001	5.79693	−19.3356
52	−10.8108	−7.11297	−13.7792	117	−12.6028	8.41981	−19.3352
53	−12.1598	−4.42846	−13.7767	118	−10.7193	10.71902	−19.3342
54	−12.8549	−1.50563	−13.7753	119	−8.42177	12.60771	−19.3335
55	−12.8577	1.500568	−13.7738	120	−5.80044	14.01112	−19.3328
56	−12.1677	4.426843	−13.7729	121	−2.95581	14.87613	−19.3325
57	−10.8205	7.115429	−13.7712	122	0.00027	15.16902	−19.3325
58	−8.88821	9.421229	−13.768	123	2.958304	14.87903	−19.3313
59	−6.47553	11.21985	−13.7663	124	5.80506	14.01674	−19.3316
60	−3.71354	12.41274	−13.765	125	8.42962	12.61466	−19.3321
61	−0.75231	12.93706	−13.765	126	10.72956	10.72666	−19.3319
62	2.249207	12.7629	−13.7632	127	12.61657	8.42569	−19.3323
63	5.13327	11.90178	−13.7647	128	14.01705	5.80143	−19.3323
64	7.740772	10.39582	−13.7631	129	14.87798	2.954624	−19.333
65	9.929975	8.329598	−13.7639	130	15.16616	−0.00206	−19.3338
